# Do Jointly Appointed Nursing and Midwifery Clinical Academics Provide Benefits to Patients, Individual Joint Appointees, Academic Institutions and Health and Social Care Organisations? A Scoping Literature Review

**DOI:** 10.1002/nop2.70227

**Published:** 2025-05-08

**Authors:** Prisca Kaunda, Hugo C. van Woerden, Ben Fitzpatrick, Vivien Coates

**Affiliations:** ^1^ Ulster University School of Nursing and Paramedic Science Derry/Londonderry United Kingdom; ^2^ University of the Highlands and Islands Inverness United Kingdom

**Keywords:** clinical academic, health benefits, joint appointments, midwifery, nursing

## Abstract

**Aim:**

This review aimed to assess the evidence of benefit from Nurses and Midwives' Clinical Academic (NMCA) appointments and establish the value of their contribution to the key stakeholders: patients, the individual joint appointees, academic institutions and health and social care organisations.

**Background:**

Jointly appointed clinical academic posts for nurses and midwives are rare, making up less than 0.1% of the workforce in the UK.

**Design:**

A scoping review.

**Methods:**

Conducted following the Joanna Briggs Institute (JBI) Methodology for Scoping Reviews.

**Data Sources:**

ProQuest, SCOPUS, MEDLINE Ovid, CINAHL Ultimate and British Library EThOS were searched for English‐language publications from January 2013 to December 2023.

**Results:**

Thirteen papers met the inclusion criteria. Key themes were the introduction of effective care guidelines and interventions, shared decision‐making in care and research, individual professional growth and development, motivation and job satisfaction, improved clinical–academic partnerships and research advancement.

**Conclusion:**

There is emerging evidence of significant benefits from clinical academic posts in nursing and midwifery; studies have generally been qualitative, focusing less on quantitative approaches.

**Implications for the Profession and/or Patient Care:**

This study demonstrates potential benefits to both the nursing/midwifery profession and patients, particularly regarding the generation of new knowledge and provision of quality care.

## 1 Introduction

Joint appointments enable health professionals to work across the service‐academic divide providing an innovative approach to accelerate the translation of research to patient benefits and population health (Academy of Medical Sciences 2020). These posts are commonly implemented as clinical‐academic roles between Higher Education Institutions and Health and Social Care organisations (Association of UK Universities and Hospitals 2019). In this context, ‘clinical‐academic joint appointments’ may be defined as “separate organisations developing shared staffing arrangements to recruit a health professional to work for both organisations to achieve shared goals” (NHS Scotland 2014 p3).

Individuals holding these joint posts are known as clinical academics and they are health and care professionals who work in Higher Education Institutions (HEI) in a teaching and/or research role, as well as working clinically in the National Health Service (NHS) (NHS Scotland 2014). This role also requires them to hold a clinical qualification and maintain a professional registration, and they provide a combination of research, clinical practice and education services (Newington et al. 2020). They concurrently engage in clinical and academic activities, provide clinical leadership and conduct research (Council of Deans 2018).

In Nursing and Midwifery, clinical academic roles are envisaged to lead and contribute to new knowledge about care and treatment through research, improving patient outcomes by providing evidence‐based healthcare, enhancing quality, safety and effectiveness, supporting research and capacity building (Coad et al. 2019). There is some evidence that research‐active hospitals are associated with an increase in patients' confidence in staff, and patients are better informed about their conditions and treatment, which may contribute to positive health outcomes (Jonker and Fisher 2018; Jonker et al. 2020). NMCAs could potentially contribute to this hospital‐level effect.

However, Nursing and Midwifery Clinical Academic (NMCA) appointments make up less than 0.1% of the workforce in the UK. It is vital to understand why such positions are commissioned so infrequently and to determine whether this is due to a lack of perceived benefits from NMCA posts for relevant stakeholders. Therefore, this scoping review was conducted to map the available evidence of the benefits of NMCA appointments to the key stakeholders. The review also aimed at identifying additional areas for further research on the topic.

### 1.1 The International Picture of Clinical Academics in Nursing and Midwifery

In Australia, the title clinical academic role is recognised as the Clinical Professor with formal posts financially supported by a healthcare organisation and higher education institute (Carrick‐Sen and Moore 2019). Although this is the case, a formal national research training pathway is not available and has had little development (Carrick‐Sen and Moore 2019). Similarly, in China, nurses and midwives are involved in research activities, but the use of clinical academic roles is not yet fully established. Efforts are being employed to increase research capacity, for example, through the development of the clinical research nurse training programme (Sun et al. 2023). In Nordic countries, research‐active nurses and midwives are recognised, with the current focus on PhD‐trained professionals within the clinical setting, but there is no formal training pathway for aspiring clinical academics (Elgaard Sørensen et al. 2019). In the USA, clinical academic posts for nurses and midwives, such as clinical professors, are available. The main career pathway for PhD‐prepared nurses is in academia, with clinical academic careers encouraged by programmes such as the Magnet Recognition Programme (Sanders et al. 2020).

### 1.2 Training and Career Pathways for NMCAs

There is some evidence that the establishment of Doctor of Nursing Practice or Doctor of Philosophy programs increases clinical academic positions (Vance et al. 2020), but limited evidence exists regarding how well career pathways are developed. In the UK, while there is a well‐established clinical academic career pathway for doctors and dentists, the pathways for nurses and midwives remain underdeveloped. To address this gap, nurses, midwives and allied health professionals have been incorporated into the clinical academic careers framework under the Health Education England (HEE)/National Institute for Health and Care Research (NIHR) Integrated Clinical Academic Programme (Health Education England 2018). The framework offers five distinct programmes at various career stages, including internships, pre‐doctoral clinical and practitioner academic fellowships, doctoral clinical and practitioner research fellowships, clinical and practitioner lectureships, and senior clinical and practitioner lectureships. Baltruks and Callaghan (2018) argue that although funding opportunities for research exist for nurses and midwives, these often do not lead to a sustainable clinical academic career. Other parts of the UK, such as Northern Ireland, lack a systematic approach to clinical academic research careers for nurses and midwives, although researchers can seek research project funding from other sources.

### 1.3 Barriers to Nursing and Midwifery Clinical Academics Appointments

Several studies have explored the barriers and facilitators for planning, establishing and retaining the NMCA joint appointments. Systematic reviews indicate that some of the perceived barriers include a lack of visible national policy, guidance, or incentives for local education and training boards, lack of compatibility between academic and service salaries with no clinical academic pay scale, lack of management support, limited funding, inadequate availability of posts, perceived conflict with service delivery demands and lack of realistic expectations between employers (Association of UK Universities and Hospitals 2019; Avery et al. 2022). This evidence suggests that the presence or adequacy of these factors could promote the posts. Additionally, it has been noted that healthcare leaders are required to promote a culture in which such posts thrive, dispelling misconceptions about research, collaborating with funders and employing transformative approaches (Aspinall et al. 2024). However, this body of literature has largely assumed the benefits of such appointments rather than subjecting them to critical scrutiny. This study is designed to address that gap.

### 1.4 Related Research on This Topic

Although not quite the same as benefits, a 2021 systematic review by Newington et al. assessed the impacts of clinical academic activity by healthcare professionals outside medicine across seven themes: impacts for patients, impacts for service provision and workforce, impacts to research profile, culture and capacity; economic impacts; impacts on staff recruitment and retention; impacts to knowledge exchange; and impacts to the clinical academic (Newington, Alexander, and Wells 2021). However, this review did not primarily focus on nurses and midwives, who form an important and distinct professional group within healthcare and who are the focus of this paper. The review concluded that further work is needed to explore standardised methods of capturing the research impact of clinical academics outside medicine.

### 1.5 Study Aim

This scoping review aimed to assess the evidence of benefit from NMCA appointments and establish the value of their contribution to the key stakeholders: patients, the individual joint appointees themselves, academic institutions and health and social care organisations.

## 2 Methods

This scoping review was conducted according to the Joanna Briggs Institute (JBI) methodology, registered on Open Science Framework and is accessible from https://osf.io/f9k53OSF.

### 2.1 Inclusion and Exclusion Criteria

The review included primary research studies published in English from 2013 to 2023 that assessed the benefits of Nurses' and Midwives' Clinical Academic posts. The ten years were considered adequate for identifying relevant research and current developments on the topic. The review excluded master's dissertations, conference reports and joint posts that were not between health and social care organisations and universities. Research related to other professional groups, such as medicine and dentistry, was also excluded. The inclusion and exclusion criteria are summarised in Table 1.

**TABLE 1:** Inclusion and Exclusion Criteria.

Criteria	Specific criteria
Inclusion	Published research papers or PhD theses. Publication in the English language Publication between 1 January 2013 and 31 December 2023 Clinical academic post linked to a recognised university and a health or social service organisation. Posts that relate to nurses or midwives Intervention studies; observational studies including cohort studies; case series; qualitative studies. Commentaries and expert opinions were only included if they incorporated some new, original data.
Exclusion	Master's dissertations Only medicine and dentistry joint appointments Only allied health professional joint appointments Studies where any NMCA‐specific component could not be identified.

### 2.2 Search Method

The reviewers searched the following databases: ProQuest, SCOPUS, MEDLINE Ovid and CINAHL Ultimate. A brief search was also conducted in the British Library EThOS. Additionally, backward chaining was used to identify additional studies.

The following combination of search terms was used to search the databases (Clinical‐academic) or “Clinical academic” or (clinical‐academician) or “Clinical academician” or (Clinical‐lectur*) or, “Clinical lectur*” or (clinical‐professor) or “Clinical professor” or (Nurse‐scientist) or “Nurse scientist” or (clinical research nurse consultant) or “Clinical research nurse consultant” AND (Nur*) or (“Nursing”) or (Midwife*) or (“Midwifery”) AND (Benefits) or (Advantages) or (Rewards) or (Improvement*) or(“Positive impact”).

The searches were conducted in February 2024 and were repeated in August 2024 before submission for publication to identify new relevant studies.

### 2.3 Selection Process

**FIGURE 1:**
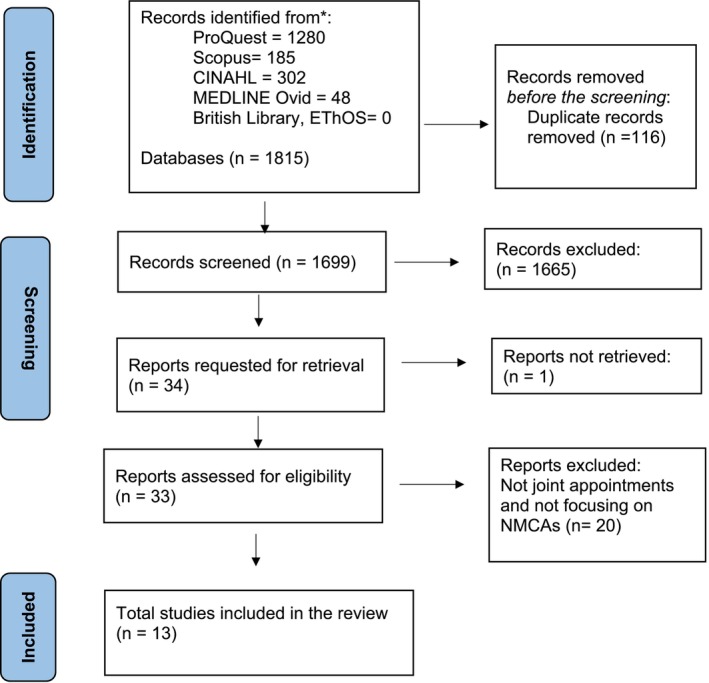
Study selection process.

The study selection process used three phases: identification, screening and eligibility, as illustrated by the Preferred Reporting Items for Systematic Reviews and Meta‐analyses extension for scoping reviews (PRISMA‐ScR) flow diagram (Figure 1). During the identification phase, the search strategy was applied to the databases to identify relevant articles, followed by the removal of duplicates. The screening phase involved checking the titles and abstracts of articles to exclude irrelevant articles. In the eligibility phase, full‐text articles were read for relevance to this review. The screening of titles and abstracts and full‐text checks were conducted by two reviewers (PK, VC, HvW). Where disagreement existed between the reviewers, a third reviewer was consulted (VC, HvW).

### 2.4 Data Abstraction and Synthesis

Data extracted included the author's name, year of publication, country of publication, publication type and findings, as summarised in Table 2. Subsequently, a narrative synthesis of the identified papers was conducted.

**TABLE 2:** Summary of data extracted.

Author	Title	Setting	Benefits for patients/clients or their families	Benefits for individual clinical academic	Benefits for academic institution	Benefits for healthcare organisations
(Oostveen et al. 2017)	Combining clinical practice and academic work in nursing: A qualitative study about perceived importance, facilitators and barriers regarding clinical academic careers for nurses in university hospitals	Netherlands	Clinical nurse academics delivered more clinically relevant education	Promoting clinical academic careers positively affect personnel outcomes because the pathway provides nurses with a chance to obtain a more challenging and enjoyable job	Clinical academic nurses establish more clinically relevant research questions and might implement research results more successfully	NMCAs positively influenced the hospital's image as an attractive employer for highly motivated and talented nurses
(Paton et al. 2022)	Journey to a new era: An innovative academic‐practice partnership	Memphis‐ USA		These cross‐organisational resources support the advancement in their respective areas of specialty	Strengthened the academic‐clinical organisation partnership More accessible resources, such as clinical sites	Strengthened the academic‐clinical organisation partnership More accessible resources, such as the university library
(Lee 2023)	A Blueprint Guide: Clinical Academia	England		Remained clinically relevant by retaining an area of practice improving confidence and credibility Working in a dual role held a high standard of teaching, knowledge base and clinical practice which is considered a personal motivator and provides exceptional satisfaction		
(Westwood et al. 2018)	Building clinical academic leadership capacity: sustainability through partnership	England‐UK			Connected NHS Trust Boards and senior management to our strategy to support integration within the clinical areas Developed clinical academic capacity and capability Developed a Doctoral Training Centre for health professionals	Connected NHS Trust Boards and senior management to our strategy to support integration within the clinical areas
(Trusson et al. 2019)	A mixed‐methods study of challenges and benefits of clinical academic careers for nurses, midwives and allied health professionals	England	Improved patient outcomes and experiences. Increase in participation in care and patient satisfaction	Job satisfaction, Increased awareness of research, enhanced skills and sense of achievement. The clinical academic pathway presented opportunities for career progression. Multiple academic journal articles and conference presentations	Multiple academic journal articles and conference presentations enabling worldwide dissemination of research	Clinical academic careers could address current issues with recruitment and retention The potential for substantial savings. For example, one participant's intervention removed the need for GPs' referral for physiotherapy, potentially saving multimillion pounds across the NHS
(Newington, Alexander, and Wells 2021)	Impacts of clinical academic activity: qualitative interviews with healthcare managers and research‐active nurses, midwives, allied health professionals and pharmacists	UK	Increased involvement of patients in evidence‐based treatment decision‐making; improved problem solving; and greater awareness of the burden on caregivers	Exposure to different research methodologies and research opportunities, practical guidance and becoming connected with like‐minded individuals. The perceived positive reputation largely stemmed from showcasing clinical academic successes and opportunities. Academic outputs, such as publications and presentations as well as developing a national standing, with individuals being contacted to provide clinical and research expertise		Clinical academic activity was perceived to contribute to beneficial cultural changes relating to the provision and delivery of clinical care and research engagement Clinical academics are exemplars in their teams, highlighting the positive contributions they were making to the local research culture This positive reputation was also perceived to contribute to improved recruitment of clinical staff to the Trust and the retention of existing staff
(Sanders et al. 2022)	Embedding post‐doctoral clinical academic careers in practice: The St Bartholomew's Hospital model	London‐UK		Consideration of individual preferences/career plans, accommodate longer‐term flexible clinical/academic time splits, Provide opportunity and support at all stages of career (including early career) and consider service development needs Provided regular research training/engagement programs to reduce ‘research fear’ and increase research skills	Regular research training/engagement programs to reduce ‘research fear’ and increase research skills Working in partnership with clinical and general managers to support predoctoral and doctoral training and role	Built clinical academic infrastructure Increased visibility of research across the organisation
(Pattison et al. 2022)	Florence Nightingale's legacy for clinical academics: A framework analysis of a clinical professorial network and a model for clinical academia	UK	Impact national level (NICE, national policy, guidelines) as well as locally	Recognised research experts as well as clinical academic leaders	High‐level trust and university influence in both practice and research Ability to ensure research is a high trust priority and impact on practice is a high priority	Ability to impact at the national level (NICE, national policy, guidelines) as well as locally (leading/developing clinical services) Recognised research experts as well as clinical academic leaders. Developing clinical academics of the future; shaping the national agenda of practice and research clinical areas of expertise
(Roddam et al. 2019)	Developing clinical academic researchers: Insights from practitioners and managers in nursing, midwifery and allied health	UK	Enhanced engagement of patient groups in research	Developed a self‐driven, resourceful approach, satisfying their intellectual curiosity and passion for research, while continuing to work in clinical practice	Clinical academics contribute to and lead research studies that address questions that are grounded in genuine clinical priorities and perspectives	Research could potentially improve cost‐effectiveness as well as clinical effectiveness
(Weber et al. 2022)	A clinical‐academic partnership to develop a family management intervention for parents of preterm infants	USA‐ Cincinnati	Effective and acceptable interventions	This clinical‐academic nurse has mentored and educated dozens of staff (physicians, nurses and therapists) in research and QI Served on several Department leadership providing a much‐needed nursing perspective to the Fellows' family‐centred research projects	Accelerated synergy with partners Research review barriers were removed, which expedited execution of the research provided continuity of personnel to the research and served as basic infrastructure for new research projects	Enhanced capacity to conduct research and QI Research review barriers were removed, which expedited execution of the research provided continuity of personnel to the research and served as basic infrastructure for new research projects
(Lauck et al. 2022)	Promoting Cardiovascular Nursing practice and research: A model for a university joint appointment	Canada		Participation as a research mentor for the Providence Health Care Practice‐Based Research Challenge, a long‐standing program that provides competitive grants to enable point‐of‐care staff to learn how to design and implement a research project Membership in the Cardiovascular Nursing Professorship in the Division of Cardiology and the organisation's research institutes		Raised physicians' and other scientists' awareness of the contributions of nursing, fostered research collaborations and promoted the organisation's pursuit of patient‐centred multidisciplinary research and practice a university role provides access to collaboration, resources and infrastructure, and scholarly collegiality

## 3 Results

### 3.1 Search Findings

The Preferred Reporting Items for Systematic Reviews and Meta‐Analyses extension for Scoping Reviews (PRISMA‐ScR) flow diagram was used to present the study selection process, as shown in Figure 1. Initially, 1815 articles were identified and 116 duplicates were removed. 1699 titles and abstracts were screened and 34 were included for retrieval. Of the 34 studies, 33 were retrieved for full‐text assessment. Of the 33 studies, 20 were removed as they did not focus on joint appointments and did not focus on nurses and midwives. 13 studies met the inclusion criteria and were included in the review. No additional studies were identified through backward chaining.

### 3.2 Characteristics of the Included Studies

The scoping review included thirteen (*n* = 13) research papers published in English, as summarised in Table 3. Most of the studies were conducted in the UK (*n* = 9), with the other countries represented as follows: USA (*n* = 2), Canada (*n* = 1) and Netherlands (*n* = 1). The studies were published between the years of 2017 to 2023. All the studies investigated nurses' and midwives' clinical academic appointments between universities and hospitals. Most of the included papers were qualitative studies (*n* = 11); among these, there were case studies (*n* = 8), in‐depth interviews/focus groups (*n* = 2) and workshops (*n* = 1). One study used a mixed‐method design combining an online survey and in‐depth interviews and the final study used a quantitative approach involving a retrospective review of records.

**TABLE 3:** Characteristics of the included studies.

Type of evidence source	Published research (*n* = 13)
Language	English (*n* = 13)
Setting	UK (*n* = 9) USA (*n* = 2) Canada (*n* = 1) Netherlands (*n* = 1)
Year of publication	2017–2023
Type of clinical academic post	NMCA, between university and hospital (*n* = 13)
Type of Study	Quantitative (*n* = 1) Qualitative studies (*n* = 12) Case studies (*n* = 8) In‐depth interviews/focus group (*n* = 2) Workshops (*n* = 1) Mixed methods (*n* = 1)

### 3.3 Findings of the Included Studies

The included studies considered several benefits of NMCA posts. We categorised these as benefits for patients/clients and families, individual clinical academics, academic institutions and healthcare organisations (see Table 4).

**TABLE 4:** Benefits from Nurses' and Midwives' Clinical Academic Appointments.

Category	Benefits
Patients/clients and families	Introduction of effective care guidelines and interventions Receiving quality care Involvement of patients and caregivers in care decision‐making Involvement of patients and caregivers in research
Individual clinical academic	Individual professional growth and development Motivation and job satisfaction Clinical confidence and credibility
Health and Social Care organisations & Academic institutions	Improved Clinical‐Academic partnerships Improved Health and Social Care organisation reputation Research advancement

### 3.4 Benefits for Patients/Clients and Families

There was some evidence of improvement in care delivery as a benefit of NMCA posts for patients/clients and families. Five studies reported that NMCAs introduced effective guidelines, thereby improving the quality of care provided (Oostveen et al. 2017; Trusson et al. 2019; Carter et al. 2020; Weber et al. 2022; Pattison et al. 2022). Three out of the five studies reported that NMCAs developed education, exercise and neonatal care interventions that were more efficient and acceptable to patients and families (Oostveen et al. 2017; Trusson et al. 2019; Weber et al. 2022). Besides, NMCAs were recognised for their contribution towards national and local policies and guidelines development leading to the provision of quality care (Pattison et al. 2022).

Another four studies stated that NMCAs facilitated patient/family involvement in research, which was perceived as a contribution to other patients' care (Trusson et al. 2019; Roddam et al. 2019; Weber et al. 2022; Spring et al. 2023). Furthermore, two studies recounted that NMCAs enhanced patient/caregiver involvement in care decision‐making, which increased acceptability and satisfaction with care (Trusson et al. 2019; Newington, Alexander, and Wells 2021).

### 3.5 Benefits for Individual Clinical Academic

Individual NMCA professional growth and development was heavily emphasised as a benefit in the included studies (Oostveen et al. 2017; Trusson et al. 2019; Roddam et al. 2019; Carter et al. 2020; Newington, Alexander, and Wells 2021; Paton et al. 2022; Sanders et al. 2022; Pattison et al. 2022; Weber et al. 2022; Lauck et al. 2022; Lee 2023; Spring et al. 2023). Five studies reported that NMCAs experienced an advancement in professional outcomes, credibility and confidence due to the availability of career pathways and essential resources for professional growth (Oostveen et al. 2017; Trusson et al. 2019; Sanders et al. 2022; Lee 2023; Spring et al. 2023).

Four other studies stated that NMCAs were more motivated and had improved job satisfaction when meeting the challenges of these demanding posts (Oostveen et al. 2017; Trusson et al. 2019; Roddam et al. 2019; Lee 2023). Furthermore, two studies recounted that NMCAs increased their knowledge and skills in the areas of expertise, raising their professional profiles (Paton et al. 2022; Lee 2023). Senior NMCA professional profiles led to improved visibility and a positive reputation, as well as advancing the mentoring of others, in seven studies (Carter et al. 2020; Newington, Wells, et al. 2021b; Paton et al. 2022; Pattison et al. 2022; Weber et al. 2022; Lauck et al. 2022; Spring et al. 2023).

### 3.6 Benefits for Healthcare Organisations

There was some evidence that healthcare organisations also benefited from NMCA posts. Eight studies reported that NMCA posts improved clinical–academic partnerships through collaborations in research, quality improvement projects and resource mobilisation, enhancing the delivery of quality care (Westwood et al. 2018; Carter et al. 2020; Paton et al. 2022; Sanders et al. 2022; Weber et al. 2022; Lauck et al. 2022; Spring et al. 2023). The healthcare organisations had more access to resources for research, enhancing research engagement and a positive research culture as demonstrated by six studies (Newington, Alexander, and Wells 2021; Paton et al. 2022; Sanders et al. 2022; Pattison et al. 2022; Weber et al. 2022; Lauck et al. 2022). Three studies reported that NMCA had contributed to improved healthcare organisations' reputations and were perceived as attractive employers for talented nurses and midwives (Oostveen et al. 2017; Newington, Alexander, and Wells 2021; Spring et al. 2023). In addition, the delivery of a more cost‐effective service was reported to have been achieved through NMCA appointments in two studies (Roddam et al. 2019; Trusson et al. 2019).

### 3.7 Benefits for Academic Institutions

Studies that were included also reported the benefits of NMCA appointments to academic institutions. Eight studies reported that NMCAs facilitated research advancement in the Universities through capacity building, development and dissemination of relevant research (Oostveen et al. 2017; Westwood et al. 2018; Trusson et al. 2019; Roddam et al. 2019; Carter et al. 2020; Sanders et al. 2022; Pattison et al. 2022; Spring et al. 2023). Academic institutions also achieved improved academic‐practice partnerships through NMCA appointments by prioritising research and its impact on healthcare, as described by five studies (Westwood et al. 2018; Paton et al. 2022; Pattison et al. 2022; Sanders et al. 2022; Weber et al. 2022). However, little evidence was reported regarding improved clinical teaching by subject experts in academic institutions, specifics about increased high‐calibre papers, or increased grant income.

## 4 Discussion

### 4.1 Main Findings

Nurses and midwives are a minority among clinical academic health professionals (Baltruks and Callaghan 2018), highlighting the need to identify and quantify the benefits they provide. This paper reviewed and presented the benefits of NMCAs, as reported in the literature. The benefits were categorised for patients, individual clinical academics, healthcare organisations and academic institutions.

#### 4.1.1 Benefits for Patients/Clients and Families

For patients related benefits, NMCAs enhance the delivery of quality care by developing effective guidelines as well as increasing patient participation in care decision‐making and research (Oostveen et al. 2017; Trusson et al. 2019; Weber et al. 2022). This promotes the implementation of evidence‐based care, warranting good health outcomes. These results align with previous research in which integrating evidence‐based practices in clinical settings improved patient health outcomes (Baptiste et al. 2022) and research‐active hospitals were associated with providing better quality information and overall inpatient experience (Jonker et al. 2020). Moreover, a notable correlation was reported to exist between increased research and reduced mortality rates (Jonker and Fisher 2018). However, additional research is required to evaluate the impact of implementing such interventions.

#### 4.1.2 Benefits for Individual Clinical Academic

Regarding individual NMCA, the joint posts have the potential to advance professional growth and development. This is attributed to advancement in knowledge and skills in their field (Paton et al. 2022; Lee 2023) ultimately achieving credibility and confidence in clinical practice (Newington, Alexander, and Wells 2021; Lee 2023). This contrasts with academic‐only positions in universities, where NMCA's competence has occasionally been questioned in clinical practice (Oostveen et al. 2017). NMCAs often face conflicts between research and clinical priorities, complicating their combined roles (Bradbury et al. 2021). Despite these challenges, many NMCAs find job satisfaction and motivation in the demanding nature of their roles (Oostveen et al. 2017; Trusson et al. 2019; Roddam et al. 2019; Lee 2023). Moreover, though challenging, joint roles increase nurses' and midwives' motivation for their work and provide the benefit of not choosing between academic work and clinical practice (Association of UK Universities and Hospitals 2019). Such posts may also have the potential to improve staff commitment to their role within an organisation and clinical community.

#### 4.1.3 Benefits for Health and Social Care Organisations and Academic Institutions

NMCAs hold significant potential to strengthen partnerships between healthcare organisations and universities, leading to better utilisation of research (Academy of Medical Sciences 2020) through collaborative research efforts, quality improvement projects and resource mobilisation. Having NMCAs in healthcare organisations improves their reputation as they are known to represent high‐calibre health professionals (Oostveen et al. 2017; Newington, Alexander, and Wells 2021; Spring et al. 2023). This could potentially contribute to increased staff retention, addressing the widespread shortage of nurses and midwives in general, as well as for NMCAs (Association of UK Universities and Hospitals 2019). Additionally, guidelines and interventions developed by NMCAs have been shown to replace inefficient practices (Roddam et al. 2019; Trusson et al. 2019) resulting in more cost‐effective services. However, additional evidence is needed to fully evaluate the cost‐effectiveness of these interventions. There was less evidence than anticipated for improved clinical teaching by subject experts in higher educational institutes, increased high‐calibre papers, or increased grant income for universities.

### 4.2 Comparison With the Wider Literature

A previous discursive review of approaches for establishing and sustaining Clinical Academic Partnerships (CAPs) supports our findings that integrating evidence‐based practices in clinical settings improves patient health outcomes and satisfaction (Baptiste et al. 2022). Similarly, a systematic qualitative review on the impact of clinical academic roles outside medicine reported increased patient involvement in clinical decision‐making and improved staff recruitment and retention (Newington, Wells, et al. 2021a). However, it remains unclear whether senior decision‐makers in healthcare organisations value such benefits, as they do not appear to be closely related to the performance metrics by which these organisations are assessed. For example, although clinical academics provide healthcare organisations with greater access to resources for research, ultimately leading to the implementation of evidence‐based care (Newington, Alexander, and Wells 2021; Paton et al. 2022; Sanders et al. 2022; Pattison et al. 2022; Weber et al. 2022; Lauck et al. 2022), this may be seen as having little value to senior healthcare managers, who are primarily assessed by financial and operational deliverables (Academy of Medical Sciences 2020).

It is worth noting that CAPs are commonly used to promote translational research in other settings such as the USA and Canada, where CAPs are formulated between academic and health organisations to promote research. Although the approach is different, several case studies on CAPs have demonstrated similar benefits to joint appointments, such as enhanced clinical academic collaboration (Paton et al. 2022), improved patient health outcomes (Clevenger and Cellar 2018) and health system innovation (Phillips et al. 2019) It would be interesting to understand why clinical academic partnerships are more prevalent in some settings than joint appointments.

### 4.3 Strengths of the Review

This review provides evidence of the benefits of NMCAs to relevant stakeholders. This evidence is expected to assist individual NMCAs, employers and funding organisations in having a better understanding of the value of these posts through the presented benefits. The review processes followed evidence‐based guidelines intended to capture relevant research over the last decade. The study selection process was conducted by two reviewers and conflicts were resolved by involving a third reviewer, enhancing the study's validity. The benefits identified in this review are also categorised in a way that aligns with settings for the potential implementation of our findings.

### 4.4 Limitations of the Review

Stakeholders should take the following considerations when interpreting or using the findings of this review. There were relatively few studies that fit the inclusion criteria as the focus was on joint nursing and midwifery appointments. The search strategy was limited to studies reported in English, which may have led to the exclusion of other benefits documented in non‐English studies. No relevant PhD theses were found through the British EThOS, although its functionality was limited. Quality assessment and impact capture tools were not used in this review, in keeping with guidance for scoping reviews, as our aim was restricted to mapping the available evidence on the topic.

### 4.5 Recommendations for Further Research

Further work is needed to identify the benefits that are most valued by those who are responsible for initiating, authorising and funding NMCA posts, perhaps drawing on lessons from success in other professional groups. A collection of more quantitative data to clarify which benefits are most valued by such key stakeholders, for example, improved patient outcomes (reduced readmission rates, shorter hospital stays and higher patient satisfaction scores), reduced costs, increased staff retention and enhanced organisational rankings is necessary. Additional qualitative data, such as exploring staff satisfaction, improved collaboration and professional development, may also help create a robust case for further joint appointments. Further research could also explore the economic impact, whether NMCAs replace or reduce waste or more expensive practices, leading to cost savings for healthcare organisations.

### 4.6 Implications for Policy and Practice

This study provides some evidence for the value of NMCA as demonstrated by the identified benefits. To convince funders to invest in these strategic posts, greater evidence perhaps needs to be gathered on the benefits to those who resource such posts. Evidence of the benefits could also lead to governmental policies that allocate more funding and resources to support NMCA positions including career pathways, grants, fellowships and funding for relevant research projects.

## 5 Conclusion

This review was set out to map evidence of the benefits of NMCA appointments to relevant stakeholders. The benefits of NMCAs include improvement in the quality of care provided to patients/families, professional growth and development for individual NMCAs, enhanced clinical academic collaboration and research advancement for academic institutions and health and social care organisations. The findings can help relevant stakeholders understand the contributions of NMCAs to health care, thereby improving their planning, recruitment and retention of such posts.

## Author Contributions

**Prisca Kaunda:** literature retrieval and screening; data curation; data synthesis and analysis; writing review and editing. **Hugo C. van Woerden:** topic selection; writing review and editing. **Vivien Coates:** topic selection; writing review and editing. **Ben Fitzpatrick:** review of final paper.

## Disclosure

*Reporting Method*: PRISMA checklist for scoping reviews. *Trial and Protocol Registration*: 16 February 2024 https://osf.io/f9k53OSF.

## Conflicts of Interest

The authors declare no conflicts of interest.

## Supporting information


DataS1.

DataS2.

DataS3.

DataS4.

## Data Availability

All data of this study's findings are included in this article.
