# Supplementary material for: Do Jointly Appointed Nursing and Midwifery Clinical Academics Provide Benefits to Patients, Individual Joint Appointees, Academic Institutions and Health and Social Care Organisations? A Scoping Literature Review
Source: Nurs Open. 2025 May 8;12(5):e70227. doi: 10.1002/nop2.70227 (PMC12061839; doi:10.1002/nop2.70227)
Supplement: Supplementary file 3 — DataS3. [file NOP2-12-e70227-s003.pdf]

DO JOINTLY APPOINTED NURSING AND MIDWIFERY CLINICAL ACADEMICS PROVIDE BENEFITS TO PATIENTS, INDIVIDUAL JOINT APPOINTEES, ACADEMIC INSTITUTIONS, AND HEALTH AND SOCIAL CARE ORGANISATIONS? A SCOPING LITERATURE REVIEW

**SUMMARY OF DATA EXTRACTION**						
**REFERENCE ID**	**TITLE**	**SETTING/ COUNTRY**	**Benefits for Patients/clients or their families**	**Benefits for an Individual clinical academic**	**Benefits for an Academic institution**	**Benefits for Healthcare organisations**
(Oostveen *et al.* 2017)	Combining clinical practice and academic work in nursing: A qualitative study about perceived importance, facilitators and barriers regarding clinical academic careers for nurses in university hospitals	Netherlands	• Clinical nurse academics delivered more clinically relevant education	• Promote clinical academic careers positively affects personnel outcomes because the pathway provides nurses with a chance of obtaining a more challenging and enjoyable job	• clinical academic nurses establish more clinically relevant research questions and might implement research results more successfully	• NM clinical academics positively influenced the hospitals' image as an attractive employer for highly motivated and talented nurses:
(Paton *et al.* 2022)	Journey to a new era: An innovative academic-practice partnership	Memphis-USA		• These cross-organizational resources support the advancement in their respective areas of specialty.	• Strengthened the academic-clinical organization partnership<br>• More accessible resources such as clinical sites	• Strengthened the academic-clinical organization partnership<br>• More accessible resources such as the university library.

9(Lee 2023)	A Blueprint Guide: Clinical Academia	England		• Remained clinically relevant by retaining an area of practice improving confidence and credibility<br>• Working in a dual role held a high standard of teaching, knowledge base, and clinical practice which is considered a personal motivator and provides exceptional satisfaction		
(Westwood *et al.* 2018)	Building clinical academic leadership capacity: sustainability through partnership	England-UK			• Connected NHS Trust Boards and senior management to our strategy to support integration within the clinical areas.	• Connected NHS Trust Boards and senior management to our strategy to support integration within the clinical areas

					• Developed clinical academic capacity and capability<br>• Developed a Doctoral Training Centre for health professionals	

(Trusson *et al.* 2019)	A mixed-methods study of challenges and benefits of clinical academic careers for nurses, midwives and allied health professionals	England	• Improved patient outcomes and experiences.<br>• Increase in participation in care and patient satisfaction	• Job satisfaction,<br>• Increased awareness of research, enhanced skills, and sense of achievement.<br>• The clinical academic pathway presented opportunities for career progression.<br>• Multiple academic journal articles and conference presentations	• Multiple academic journal articles and conference presentations enabling worldwide dissemination of research	• Clinical academic careers could address current issues with recruitment and retention.<br>• The potential for substantial savings. For example, one participant's intervention removes the need for GPs' referral for physiotherapy, potentially saving 'multimillion pounds' across the NHS

(Newington *et al.* 2021)	Impacts of clinical academic activity: qualitative interviews with healthcare managers and research-active nurses, midwives, allied health professionals and pharmacists	UK	• Increased involvement of patients in evidence-based treatment decision-making; improved problem solving; and greater awareness of the burden on caregivers	• Exposure to different research methodologies and research opportunities, practical guidance, and becoming connected with like-minded individuals.<br>• The perceived positive reputation largely stemmed from showcasing clinical academic successes and opportunities.<br>• Academic outputs, such as publications and presentations as well as developing a national standing, with individuals being contacted to provide clinical and research expertise		• Clinical academic activity was perceived to contribute to beneficial cultural changes relating to the provision and delivery of clinical care and research engagement.<br>• Clinical academics are exemplars in their teams, highlighting the positive contributions they were making to the local research culture.<br>• This positive reputation was also perceived to contribute to improved recruitment of clinical staff to the Trust, and the

						retention of existing staff
(Sanders *et al.* 2022)	Embedding post-doctoral clinical academic careers in practice: The St Bartholomew's Hospital model	London-UK		• Consideration of individual preferences/career plans, accommodate longer-term flexible clinical/academic time splits,<br>• Provide opportunity and support at all stages of career (including early career) and	• Regular research training/engagement programs to reduce 'research fear' and increase research skills<br>• Working in partnership	• Built clinical academic infrastructure<br>• Increased visibility of research across the organisation

				consider service development needs<br>• Provided regular research training/engagement programs to reduce 'research fear' and increase research skills	with clinical and general managers to support predoctoral and doctoral training and role	
(Pattison *et al.* 2022)	Florence Nightingale's legacy for clinical academics: A framework analysis of a clinical professorial network and a model for clinical academia	UK	• Impact national level (NICE, national policy, guidelines) as well as locally	• Recognised research experts as well as clinical academic leaders	• High-level trust and university influence in both practice and research<br>• Ability to ensure research is a high trust priority and impact on practice is a high priority	• Ability to impact at the national level (NICE, national policy, guidelines) as well as locally (leading /developing clinical services);<br>• Recognised research experts as well as clinical academic leaders.<br>• Developing clinical academics of the future; shaping the national agenda

						of practice and research clinical areas of expertise
(Roddam *et al.* 2019)	Developing clinical academic researchers: Insights from practitioners and managers in nursing, midwifery and allied health	UK	• Enhanced engagement of patient groups in research	• Developed a self-driven, resourceful approach, satisfying their intellectual curiosity and passion for research, while continuing to work in clinical practice	• Clinical academics contribute to and lead research studies that address questions that are grounded in genuine clinical priorities and perspectives	• Research could potentially improve cost-effectiveness as well as clinical effectiveness

(Weber *et al.* 2022)	A clinical-academic partnership to develop a family management intervention for parents of preterm infants	USA-Cincinnati	• Effective and acceptable interventions	• This clinical-academic nurse has mentored and educated dozens of staff (physicians, nurses, and therapists) in research and QI.<br>• Served on several Department leadership providing a much-needed nursing perspective to the Fellows' family-centred research projects	• Accelerated synergy with partners<br>• Research review barriers were removed, which expedited execution of the research provided continuity of personnel to the research and served as basic infrastructure for new research projects	• Enhanced capacity to conduct research and QI,<br>• Research review barriers were removed, which expedited execution of the research provided continuity of personnel to the research and served as basic infrastructure for new research projects

(Lauck *et al.* 2022)	Promoting Cardiovascular Nursing practice and research: A model for a university joint appointment	Canada		• Participation as a research mentor for the Providence Health Care Practice-Based Research Challenge, a long-standing program that provides competitive grants to enable point-of-care staff to learn how to design and implement a research project,<br>• Membership in the Cardiovascular Nursing Professorship in the Division of Cardiology and the organisation's research institutes		• Raised physicians' and other scientists' awareness of the contributions of nursing, fostered research collaborations, and promoted the organisation's pursuit of patient-centred multidisciplinary research and practice.<br>• a university role provides access to collaboration, resources, and infrastructure, and scholarly collegiality

Carter *et al.* 2020	Evaluation of the joint Nurse Scientist role across Academia and practice	USA- New York	• Effective intervention s	• Co-authored manuscripts and facilitated publications<br>• Expanded the visibility and valuation of the PhD-educated nurse.	• Facilitated research across academia and practice.	• Access to academic mentorship and collaboration, resources and infrastructure, and scholarly collegiality

Ref ID 1199	Using the Making Visible the ImpaCT of Research (VICTOR) questionnaire to evaluate the benefits of a fellowship program for nurses, midwives, and allied health professionals	UK	• Enabled patient and public involvement to be meaningful and inform aspects of the study	• Enabled personal development to learn about different methodologies and gain experience in writing grants.<br>• Protected time enabled academic outputs including publications and presentations at national and international conferences.<br>• Raised staff profile so they could be involved in national initiatives	• Protected time enabled academic outputs including publications and presentation s at national and international conferences.	• Enabled relationships to be developed with research teams in HEIs.<br>• Raised staff profile so they could be involved in national initiatives and help to attract staff to work in the trust
