# Supplementary material for: Do Jointly Appointed Nursing and Midwifery Clinical Academics Provide Benefits to Patients, Individual Joint Appointees, Academic Institutions and Health and Social Care Organisations? A Scoping Literature Review
Source: Nurs Open. 2025 May 8;12(5):e70227. doi: 10.1002/nop2.70227 (PMC12061839; doi:10.1002/nop2.70227)
Supplement: Supplementary file 4 — DataS4. [file NOP2-12-e70227-s004.pdf]

Scoping Review title:	**Do jointly appointed Nursing and Midwifery Clinical Academics provide benefits to patients, individual joint appointees, academic institutions, and health and social care organisations? A scoping literature review**
Review aim:	To assess available evidence of benefit from Nurses and Midwives' Clinical Academic (NMCA) appointments and establish the value of their contribution to the key stakeholders: patients, the individual joint appointees, academic institutions, and health and social care organisations.
Review question/s:	Are there beneficial outcomes of clinical academic joint appointments in Nursing and Midwifery to patients, individual clinical academics, health organisations, academic institutions, and funding organisations?
**Results**	
Number of studies included	(n=13)
Type of evidence source (PhD thesis/published research)	Published research (n=13)
Language	English (n=13)
Setting	UK (n= 9)<br>USA (n=2)<br>Canada (n=1)<br>Netherlands (n=1)
Year of publication	2017- 2023
Type of clinical academic post	Between the University and the hospital (n=13)

Type of Study	Quantitative (n=1)<br>Qualitative studies.<br>Case studies (n=8)<br>In-depth interviews/focus group (n=2)<br>Workshops (n= 1)		
	Mixed methods (n=1)		
**BENEFITS**			
**Category**	**Benefits**	**Subthemes**	**Themes**

Patients/clients or their families	Benefits for Patients/clients or their families<br>clinical nurse academics could deliver more clinically relevant education.<br>The research undertaken by the participants could potentially make a big difference in patient outcomes and experiences.<br>Another participant had introduced a pre-surgery exercise programme which helped patients to feel involved in the process and was highly rated in a patient satisfaction survey.<br>Increased confidence in questioning practice and openly discussing with patients and colleagues if there was uncertainty over management options;<br>increased involvement of patients in evidence-based<br>treatment decision-making; improved problem<br>solving; and greater awareness of the burden to caregivers"<br><br>Ability to impact at national level (NICE, national policy, guidelines) as well as locally (leading /developing clinical services)<br>Practitioners also suggested that clinical academics could enhance engagement of patient groups in research, including recruitment to applied healthcare studies as well as consultation to develop co-designed research plans and priorities	Introduction of effective guidelines of care<br>Patient/ care givers involvement in care decision making and research	Improvement in delivery of care

	We were able to create an accessible, platform-agnostic intervention for use in neonatal intensive care, so that parents can choose when and where they learn.<br>Mothers interacting with the first prototype perceived a benefit by knowing that they were helping other NICU families to support effective family management of preterm infant care<br><br>Under the mentorship of one joint nurse scientist, for example, a clinical nurse conducted a randomized controlled trial to assess the impact of an educational video on distress among pediatric patients undergoing a magnetic resonance imaging procedure. The video was found to significantly reduce distress, has been implemented into standard clinical practice,<br>Enabled patient and public involvement to be meaningful and inform aspects of the study		

Individual clinical academic	Benefits for Individual clinical academic<br>implementing clinical academic career pathways for nurses in hospitals to promote clinical academic careers. They believed that the implementation would positively affect the personnel outcomes because the pathway provides nurses with a chance of obtaining a more challenging and enjoyable job<br>These cross-organizational resources support the advancement of APRNs in their respective areas of specialty.<br>"Remaining clinically relevant was pivotal as it allowed me to retain an area of practice where I felt confident and credible.<br>Working a dual role holds me to an incredibly high standard of teaching, knowledge base and clinical practice which I consider an advantage and personal motivator.<br>I have found clinical academia to provide me with exceptional satisfaction as I am directly invested in the teaching students receive, as I see them apply it in practice when I work alongside them clinically."<br><br>Participants reported benefits such as job satisfaction, increased awareness of research, enhanced skills and sense of achievement.<br>The clinical academic pathway had presented opportunities for career progression.	Improved professional outcomes, credibility and confidence<br><br>Increased professional advancement due to access to essential resources<br><br>Increased motivation and job satisfaction<br><br>Increased knowledge and skills<br>Improved visibility and positive reputation<br><br>Advanced to mentor others	Individual professional growth and development

	"Perceived benefits included being exposed to different research methodologies and research opportunities, practical guidance and becoming connected with like-minded individuals.<br>The perceived positive reputation largely stemmed from showcasing clinical academic successes and opportunities. This included academic outputs, such as publications and presentations as well as developing a national standing, with individuals being contacted to provide clinical and research expertise"<br>Our model uses a bespoke approach in order to be able to consider individual preferences/career plans, accommodate longer-term flexible clinical/academic time splits, provide opportunity and support for NAHP and HCS at all stages of their career (including early career) and also consider service development needs recognised research experts as well as clinical academic leaders<br>Participants who had undergone some clinical academic training reported that it had helped them to develop a self-driven, resourceful approach, satisfying their intellectual curiosity and passion for research, while still continuing to work in clinical practice.<br>This clinical-academic nurse has mentored and educated dozens of staff (physicians, nurses, and therapists) in research and QI.<br>The PI also served on several of the Department of Neonatology Fellows' Scholarship Oversight Committees, providing a much-needed nursing perspective to the Fellows' family-centered research projects		

	Participation as a research mentor for the Providence Health Care Practice-Based Research Challenge, a long-standing programme that provides competitive grants to enable point-of-care staff to learn how to design and implement a research project,"joint nurse scientists' co-authored 17 manuscripts with nurses in health systems of these, 16 (94%) were published after faculty began the joint nurse scientist position; indicating the joint nurse scientist role facilitated research across academia and practice.<br>The joint nurse scientist role has expanded the visibility and valuation of the PhD-educated nurse. Joint nurse scientists have been increasingly asked to partner in interdisciplinary research projects and health system initiatives, including serving as advisory board members to the nurse residency program and leading and/or contributing to evaluations of participating health systems' inpatient fall prevention program""Enabled personal development to learn about different methodologies and gain experience in writing grants.<br><br>Protected time enabled academic outputs from the fellowship to be completed during the 12-month period. This included publications and presentations at national and international conferences.<br>Raised staff profile so they could be involved in national initiatives"		

Academic institution	benefits for Academic institution<br>Clinical academic nurses establish more clinically relevant research questions and might implement research results more successfully<br>This model of formal faculty appointments for practicing APRNs strengthened the academic-clinical organization partnership "We have connected NHS Trust Boards and senior management to our strategy to support integration within the clinical areas.<br>This partnership has developed clinical academic capacity and capability; in particular, we have created a critical mass of Clinical Doctoral Research Fellows.<br>We have, in effect,developed a Doctoral Training Centre for health professionals to support and nurture this sector of the workforce"<br><br>Participants provided details of multiple academic journal articles and conference presentations enabling worldwide dissemination of their research<br><br>We have provided regular research masterclasses, events and bespoke research training/engagement programmes to reduce ‘research fear’ and increase research skills and awareness, as well as working in partnership with clinical and general managers to support predoctoral and doctoral training and role	Increased development and dissemination of relevant research<br><br>Improved academic-practice partnership<br><br>Improvement in research capability and capacity building	Academic -practice partnership<br><br>Research advancement

	High-level trust and university influence in both practice and research; ability to ensure research is high trust priority; impact on practice is a high priority<br>Participants also felt that clinical academics were best placed to contribute to and lead research studies that addressed questions that were grounded in genuine clinical priorities and perspectives<br>"This feed forward loop accelerated synergy between our partners, and eventually resulted in a Career Development award from the U.S. National Institutes of Health to support pilot testing of PREEMIE PROGRESS using a randomized clinical trial design.<br>Because the clinical-academic nurse and research nurses were employees of both institutions, regulatory and hospital research review barriers were removed, which expedited execution of the research, provided continuity of personnel to the research, and served as basic infrastructure for new research projects"<br><br>"joint nurse scientists' co-authored 17 manuscripts with nurses in health systems of these, 16 (94%) were published after faculty began the joint nurse scientist position; indicating the joint nurse scientist role facilitated research across academia and practice."" Protected time enabled academic outputs from the fellowship to be completed during the 12-month period. This included publications and presentations at national and international conferences. "		

Health care organisations	Benefits for healthcare organisations<br>several participants said that the implementation also positively affected the hospitals' image as an attractive employer for highly motivated and talented nurses:<br>"This model of formal faculty appointments for practicing APRNs strengthened the academic-clinical organization partnership<br>Resources that were previously unavailable or had limited availability, such as readily accessible pediatric clinical sites and access to the university library, were subsequently more accessible to CON programs and APRNs, respectively."<br><br>We have connected NHS Trust Boards and senior management to our strategy to support integration within the clinical areas<br>"At the hospital they want this Magnet status.The three domains are good clinical outcomes, patient experience and staff experience and part of (that) is having well qualified nurses. They really want to increase the academic underpinnings of nurses and have research leaders.<br>Participants highlighted how supporting clinical academic careers could address current issues with recruitment and retention:<br>Forty thousand nurses we have a national deficit of, so people can choose where they want to work. They'll be looking for organisations that are aspirational. So	Improved hospital image and influence in national policy and guidelines<br><br>Improved clinical academic partnership<br><br>Increased access to resources for research<br><br>Improved delivery and cost effectiveness of services<br><br>Increased research engagement	Health care organization reputation<br><br>Clinical academic partnership<br><br>Delivery of health services

	actually offering innovative career pathways that can intellectually challenge, but also have that direct patient care element, is going to be attractive to a lot of people (CS9 nurse/midwife).<br>The data revealed numerous examples of impact resulting from participants' clinical academic careers, including the potential for substantial savings. For example, one participant's intervention removes the need for GPs' referral for physiotherapy, potentially saving 'multimillion pounds' across the NHS, and has subsequently been recognised in the NHS long-term plan"<br>"Clinical academic activity was perceived to contribute to beneficial cultural changes relating to the provision and delivery of clinical care and research engagement. Managers named clinical academics within their teams as exemplars, highlighting the positive contributions they were making to the local research culture.<br>This positive reputation was also perceived to contribute to improved recruitment of clinical staff to the Trust, and the retention of existing staff"<br>We have built our clinical academic infrastructure in partnership with Queen Mary University of London, who actively support and deliver this for NAHP and HCS staff as they do medical staff<br>increased visibility of research across the organisation, and this, alongside the Hospital Board and growing senior nursing, medical, allied professionals		

	Ability to impact at national level (NICE, national policy, guidelines) as well as locally (leading /developing clinical services); recognised research experts as well as clinical academic leaders. Developing clinical academics of the future; shaping the national agenda of practice and research clinical areas of expertise<br>"It was also proposed that research could potentially improve cost-effectiveness as well as clinical effectiveness,"<br>"With enhanced capacity to conduct research and QI, we have been able to start new improvement projects, including the use of a parent coordinator position and a new family reading program to improve parent psychosocial well-being in our NICU.<br>Because the clinical-academic nurse and research nurses were employees of both institutions, regulatory and hospital research review barriers were removed, which expedited execution of the research, provided continuity of personnel to the research, and served as basic infrastructure for new research projects"<br>"The Cardiovascular Nursing Professorship's membership in the Division of Cardiology and the organisation's research institutes raise physicians' and other scientists' awareness of the contributions of nursing, fosters research collaborations, and promotes the organisation's pursuit of patient-centred multidisciplinary research and practice.<br>a university role provides access to academic mentorship and collaboration, resources and infrastructure,		

	and scholarly collegiality"<br>, a university role provides access to academic mentorship and collaboration, resources and infrastructure, and scholarly collegiality<br>Enabled relationships to be developed with research teams in HEIs .<br>Raised the profile of the CNMAR, which helps to attract staff to work in the trust .<br>Raised staff profile so they could be involved in national initiatives		
